# HOME CARE SOCIAL WORKERS CLAIM MEDICARE IGNORES PATIENT NEEDS

**DOI:** 10.1093/geroni/igz038.1184

**Published:** 2019-11-08

**Authors:** William D Cabin

**Affiliations:** Temple University, Philadelphia, Pennsylvania, United States

## Abstract

There is significant literature on the importance of addressing social determinants of health (SDOH) in order to improve health care outcomes. In response, the Centers for Medicare and Medicaid Services (CMS) has expanded Medicare Advantage plans ability to cover SDOH-related services. Medicare home health does not cover SDOH-related services. A literature review indicates no studies on the nature, significance, or impacts of the lack of SDOH coverage in Medicare home health. This article summarizes an initial, exploratory study to address the literature gap, based on interviews of a convenience sample of 29 home care social workers between January 2013 and May 2014 in the New York City metropolitan area. Results indicate social workers believe the lack of SDOH coverage in Medicare home health results in exacerbation of existing patient conditions; creation of new, additional patient conditions; increased home care readmissions and re-hospitalizations; increased caregiver burden; and exacerbation of patients’ mental health and substance abuse needs. Policymakers are urged to consider adding coverage of SDOH to Medicare home health primarily through expanded social work coverage.

